# Coronavirus disease 2019 infection severity among different variants in children under 2-years old in Brazil

**DOI:** 10.1016/j.clinsp.2025.100592

**Published:** 2025-02-21

**Authors:** Beatriz Martinelli Menezes Gonçalves, Rossana P.V. Francisco, Ágatha S. Rodrigues, José Carlos Soares Junior

**Affiliations:** a Department of Obstetrics and Gynecology, Universidade de São Paulo (USP), São Paulo, SP, Brazil; b Department of Statistics, Universidade Federal do Espírito Santo (UFES), Vitória, ES, Brazil

**Keywords:** COVID-19, SARS-CoV-2, Respiratory tract infections, Children

## Abstract

To analyze whether there is a significant difference in the virulence, symptoms, and outcomes of different Coronavirus Disease 2019 (COVID-19) variants in children under 2-years of age. We collected data from the *Sistema de Informação de Vigilância Epidemiológica* da *Gripe*, a nationwide Brazilian database on severe acute respiratory syndrome. The patients were classified according to four variants of concern: wild-type, gamma, delta, and omicron. The wild-type variant was defined as the baseline. A total of 11,153 patients were analyzed. The risk of presenting dyspnea (adjusted Odds Ratio[Aor = 1.20], 95 % Confidence Interval [95 % CI 1.07–1.34]) was higher in patients with gamma infection. Respiratory discomfort was more likely to be present for the omicron (Aor = 1.29, 95 % CI 1.15–1.43) and gamma (aOR = 1.26, 95 % CI 1.13–1.41) infections. Desaturation was more likely to be present for the omicron (aOR = 1.67, 95 % CI 1.50–1.86), gamma (aOR = 1.16, 95 % CI 1.43–1.79), and delta (aOR 1.41, CI 95 % 1.18–1.68) infections. Infection by the omicron variant was a protective factor for intubation (aOR = 0.78, 95 % CI 0.67–0.91) and death (aOR = 0.43, 95 % CI 0.35–0.53). Additionally, delta infection was a protective factor against death (aOR = 0.60, 95 % CI 0.43–0.85). The wild-type variant was responsible for most of the cases that evolved with death. Omicron appears to be responsible for milder symptoms than delta. Children between 1 and 6 months of age account for most cases, which is a concern because there is no vaccination coverage.

## Highlights

• The original variant was responsible for most of the cases that evolved with death, omicron seems to be responsible for milder symptoms when compared to delta.
• Children between 1‒6-months of age account for most of the cases, which concerns since there is no vaccination coverage.

## Introduction

Coronavirus Disease 2019 (COVID-19) was officially declared a pandemic by the World Health Organization as of March 2020 after its global spread and high mortality rates. Approximately 700 million cases have been confirmed, and 7 million deaths have been associated with the disease globally.^1^ Initially, children were thought to be less susceptible to infection by the virus, and their infection was associated with milder symptoms. The disease has a tremendous impact worldwide; however, few studies have analyzed the impact of COVID-19 on children under 2-years of age have been published, and insufficient information is available regarding symptoms, risk factors, intensive care unit admission, and treatment. Most therapeutic procedures and information associated with the infection have been incorporated into children through research conducted in adults. The exact number of children infected and stricken by COVID-19 seems to be underestimated because most presented with mild symptoms, and others were not offered testing at the beginning of the pandemic.^2^^,^^3^ The age gap, from newborns until 2-years of age (first 1.000 life days), is considered by the United Nations International Children's Emergency Fund and World Health Organization as a crucial period in determining children's intellectual, social, and biological development.^4^

What happens during this period defines health, even into adulthood. This phase covers 270 days of gestation until 730 days of life when the child turns 2 years old. Compared with children aged 3–11 months, children aged under 1-month (26.3 [16.1–43.1]) and those aged 1–2 months (4.7 [3.1–7.34]) have higher hospitalization risk, according to reports.^5^ However, this population has not been studied extensively in relation to COVID-19 or its Variants of Concern (VOCs).

The first case of COVID-19 was identified in Hubei, China, in December 2019 and was associated with the wild-type variant. Cases and symptoms were extremely diverse, from asymptomatic patients to critical involvement of the respiratory tract with the necessity of ventilatory and intensive care support.^6^ In children, a meta-analysis published by *Medical Virology* with a total of 48 studies and 5829 pediatric patients concluded that 20 % were asymptomatic, 33 % had mild symptoms, and 51 % presented with moderate symptoms. Fever and cough were the primary symptoms.^7^

During the pandemic, new Severe Acute Respiratory Syndrome Coronavirus-2 (SARS-CoV-2) variants emerged. In Brazil, the VOC was the gamma variant, first identified in December 2019 with high virulence, although this variant did not account for a large number of cases worldwide.^5^ The second and third VOC were delta and omicron, respectively, both described in July 2021 and responsible for the second SARS-CoV-2 infective wave.^7^ Retrospectively, it was identified that gamma was dominant from February until July 2021, delta from August until December 2021, and omicron from January 2022 until 2023 in Brazil.^6^

With the presence of these new variants and a closer look at neglected populations, such as children and adolescents, an increase in confirmed SARS-CoV-2 cases in this population has occurred.^8^

The increase in children infected by SARS-CoV-2 arose with the omicron variant, and it was initially thought that this variant could be associated with severe symptoms in this population.^3^^,^^9^ Subsequently, in comparison with the delta variant, omicron was identified as responsible for milder symptoms, although it was more infectious and had a higher spread.^9^^,^^10^ Unlike in adults, not enough is known about the impact of distinct VOC in children. Studies comparing the impact of VOC are limited to omicrons and deltas and mostly include children below 15 or 18-years of age in the same group as toddlers and younger infants.^9^, ^10^, ^11^

Vaccination was first authorized for children in June 2022, but initially, it was only available for children older than 5-years.^12^ In Brazil, vaccination for children between 6-months and 5-years of age was approved by December 2022, but it is estimated that only 25 % of children between 6-months and 4-years of age have been vaccinated with the first dose, and only 2.5 % with a second dose.^13^^,^^14^ This extremely low coverage exposes children to severe forms of the disease.^13^ Furthermore, children under 6-months old are not covered by vaccination.

This study aimed to determine whether there are significant differences in virulence, symptoms, and outcomes between different SARS-CoV-2 variants in children under 2-years of age with Severe Acute Respiratory Syndrome (SARS).

## Materials and methods

For our analysis, we used data available at the *Sistema de Informação de Vigilância Epidemiológica* da *Gripe* (SIVEP-Gripe), which is a nationwide record database for all notified cases of SARS, created in 2009 and managed by the Health Ministry of Brazil (*Ministério* da *Saúde* and the *Secretaria de Vigilância em Saúde*). In Brazil, all cases of SARS have been reported, and both public and private hospitals must report them. The database contains information regarding demographic characteristics (sex, age, race, and location); clinical features, including symptoms and comorbidities; laboratory diagnosis confirming the cause of SARS; and information regarding the need for critical care, intubation, and date of death, if applicable. Specifically, cases of SARS or deaths caused by SARS, regardless of hospitalization, were further filtered into confirmed cases of COVID-19. SIVEP-Gripe is an open database with no possibility of individual identification; therefore, according to Brazilian regulations, this study does not require prior approval from the institutional ethics board.

Children aged below 2-years with SARS caused by COVID-19 confirmed by reverse transcription polymerase chain reaction were further divided into categories of different COVID-19 variants according to the most prevalent variant at that specific time point: wild-type, gamma, omicron, and delta. In Brazil, the FIOCRUZ surveillance database is a nationwide surveillance system that determines viral prevalence using genome sequencing. We used national sequencing to determine the periods in which each variant was predominant, that is, >50 % of the sequenced cases. The cases in this study were included sequentially without gaps, following the determined inclusion criteria.^15^

Four groups of patients were created according to the predominance of the isolated variants of SARS-CoV-2: wild-type, gamma, delta, and omicron.

For the analysis, only variables that were correctly and completely reported were considered. Patients for whom the outcome of cure or death was not documented were excluded. The final result was 11,153 cases, further divided into the variants of interest: 3706 cases of the wild-type variant; 2941 gamma, 795 delta, and 3711 omicrons.

We analyzed the epidemiological characteristics (ethnicity, sex, age group, and number of comorbidities), clinical outcomes (death or cure, necessity of intensive care unit treatment, and intubation), symptoms (cough, fever, dyspnea, respiratory discomfort, desaturation, diarrhea, and vomiting), and comorbidities (cardiac, renal, hepatic, hematologic, neurologic, and pneumological). The wild-type variant was established as the baseline, and three other variants of interest (gamma, delta, and omicron) were compared. Because most published studies compare the delta and omicron variants, further analysis was conducted for symptoms and outcomes, including only these two variants of interest and considering delta as the baseline. All valid cases were analyzed for the identified variables, except for comorbidities.^16^

## Data analysis

Quantitative variables are presented as means and standard deviations. Qualitative variables are presented as absolute frequencies (n) and category percentages (%).

The Chi-Square test was used to evaluate the association between the variant groups and other qualitative variables. The Odds Ratio (OR) was considered a measure of association to compare the relative odds of the occurrence of the outcome of interest with a 95 % Confidence Interval (95 % CI). The analysis of symptoms and outcomes was further adjusted for variables with statistical significance, such as ethnicity, age, and cardiac and hepatic disease, adjusted OR (aOR), and respective CI of 95 %.

The significance level is 5 %. Analyses were performed using the *R* statistical software (R Foundation for Statistical Computing Platform, version 4.0.3).^17^

## Results

A total of 11,153 cases were analyzed, accounting for children under the age of 2-years with SARS due to COVID-19, divided according to the prevalence of VOC: 3706 cases for the wild-type, 2941 for gamma, 795 for delta, and 3711 for omicron variants. Regarding epidemiological characteristics, most of the population were male and aged between 1 and 6 months. The majority of the population was brown, except during the omicron variant period when the majority was white (Table 1) Heart and hepatic diseases seemed to have different distributions among the VOC, with both being more prevalent in the wild-type variant.

**Table 1:** Epidemiologic characteristics and comorbidities.

Epidemiologic Characteristics and Comorbidities		Variants n (%)				p-value
		Wild-type	Gamma	Delta	Omicron	
Ethnicity	Black	98/2823 (3.5 %)	71/2285 (3.1 %)	14/612 (2.3 %)	88/2858 (3.1 %)	<0.001
	Brown	1577/2823 (55.9 %)	1172/2285 (51.3 %)	296/612 (48.4 %)	1177/2858 (41.2 %)	
	Indigenous	32/2823 (1.1 %)	7/2285 (0.3 %)	9/612 (1.5 %)	18/2858 (0.6 %)	
	White	1106/2823 (39.2 %)	1013/2285 (44.3 %)	288/612 (47.1 %)	1557/2858 (54.5 %)	
	Yellow	10/2823 (0.4 %)	22/2285 (1.0 %)	5/612 (0.8 %)	18/2858 (0.6 %)	
Gender	Female	1586/3695 (42.9 %)	1277/2934 (43.5 %)	344/793 (43.4 %)	1563/3711 (42.1 %)	0.692
	Male	2109/3695 (57.1 %)	1657/2934 (56.5 %)	449/793 (56.6 %)	2148/3711 (57.9 %)	
Age	0–1 m	842/3706 (22.7 %)	586/2941 (19.9 %)	102/795 (12.8 %)	356/3711 (9.6 %)	<0.001
	>1–6 m	1248/3706 (33.7 %)	1103/2941 (37.5 %)	269/795 (33.8 %)	1430/3711 (38.5 %)	
	>6–12 m	743/3706 (20.0 %)	585/2941 (19.9 %)	179/795 (22.5 %)	996/3711 (26.8 %)	
	>12–18 m	493/3706 (13.3 %)	366/2941 (12.4 %)	129/795 (16.2 %)	514/3711 (13.9 %)	
	>18–24 m	380/3706 (10.3 %)	301/2941 (10.2 %)	116/795 (14.6 %)	415/3711 (11.2 %)	
Heart disease	217/3706 (5.9 %)	152/2941 (5.2 %)	28/795 (3.5 %)	149/3711 (4.0 %)	<0.001	
Hematologic disease	29/3706 (0.8 %)	14/2941 (0.5 %)	4/795 (0.5 %)	12/3711 (0.3 %)	0.053	
Hepatic disease	21/3706 (0.6 %)	7/2941 (0.2 %)	2/795 (0.3 %)	8/3711 (0.2 %)	0.038	
Neurologic disease	130/3706 (3.5 %)	83/2941 (2.8 %)	20/795 (2.5 %)	128/3711 (3.4 %)	0.227	
Lung disease	77/3706 (2.1 %)	52/2941 (1.8 %)	15/795 (1.9 %)	60/3711 (1.6 %)	0.515	
Renal disease	22/3706 (0.6 %)	20/2941 (0.7 %)	2/795 (0.3 %)	14/3711 (0.4 %)	0.220	

All VOC presented with fever and cough as the main symptoms. Children infected in the predominant period of delta (OR = 1.23, 95 % CI 1.01–1.50) and omicron (Aor = 1.21, 95 % CI 1.07–1.37) variants were at higher risk of presenting with fever, although after adjustments for ethnicity, age, cardiac and hepatic disease, delta's statistical significance reduced. The risk of developing dyspnea (aOR = 1.20, 95 % CI 1.07–1.34) was higher in patients infected with the gamma variant. Respiratory discomfort was more likely to be present for the omicron (aOR = 1.29, 95 % CI 1.15–1.43) and gamma (aOR = 1.26, 95 % CI 1.13–1.41) infections, and desaturation for the omicron (aOR = 1.67, 95 % CI 1.50–1.86), gamma (aOR = 1.16, 95 % CI 1.43–1.79), and delta (aOR = 1.41, 95 % CI 1.18–1.68) infections. Regarding cough, delta (aOR = 2.12, 95 % CI 1.72–2.60) and omicron (aOR = 2.13, 95 % CI 1.89–2.40) variants were mostly associated with this symptom.

Children infected with the delta variant had a 41 % greater chance of desaturation than those infected with the wild-type variant. Infection with the omicron variant may be a protective factor against diarrhea. The gamma variant was mostly associated with vomiting (aOR = 1.20, 95 % CI 1.03–1.39) (Table 2).

**Table 2:** Symptoms regarding four VOC.

Symptoms	Variants n (%)						
	Wild-type^a^	Gamma	aOR (95 % CI)	Delta	aOR (95 % CI)	Omicron	aOR (95 % CI)
Fever	2473/3295 (75.1 %)	1842/2508 (73.4 %)	0.89 (0.79–1.01)	563/714 (78.9 %)	1.089 (0.88–1.33)	2703/3339 (81.0 %)	1.21 (1.07–1.37)
Cough	2082/3178 (65.5 %)	1905/2529 (75.3 %)	1.62 (1.43–1.82)	583/719 (81.1 %)	2.12 (1.72–2.60)	2699/3305 (81.7 %)	2.13 (1.89–2.40)
Dyspnea	1808/3069 (58.9 %)	1497/2379 (62.9 %)	1.20 (1.07–1.34)	367/654 (56.1 %)	0.91 (0.77–1.09)	1791/2935 (61.0 %)	1.12 (1.01–1.25)
Respiratory discomfort	1802/3047 (59.1 %)	1515/2344 (64.6 %)	1.26 (1.13–1.41)	394/636 (61.9 %)	1.16 (0.97–1.38)	1936/2999 (64.6 %)	1.29 (1.15–1.43)
Desaturation	1230/2889 (42.6 %)	1204/2236 (53.8 %)	1.56 (1.43–1.79)	314/625 (50.2 %)	1.41 (1.18–1.68)	1559/2858 (54.5 %)	1.67 (1.50–1.86)
Diarrhea	552/2720 (20.3 %)	371/1958 (18.9 %)	0.92 (0.80–1.07)	114/554 (20.6 %)	0.95 (0.76–1.20)	438/2570 (17.0 %)	0.73 (0.63–0.85)
Vomit	484/2701 (17.9 %)	410/1971 (20.8 %)	1.20 (1.03–1.39)	104/545 (19.1 %)	1.00 (0.79–1.27)	546/2599 (21.0 %)	1.11 (0.96–1.27)

a Baseline/aOR, OR adjusted for ethnicity, age, and cardiac and hepatic diseases.

Regarding outcomes: being infected by the omicron variant was a protective factor for evolving with intubation (aOR = 0.78, 95 % CI 0.67–0.91) and death (aOR = 0.43, 95 % CI 0.35–0.53). Additionally, delta infection was identified as a protective factor against death (aOR = 0.60, 95 % CI 0.43–0.85). For the wild-type variant, 10.2 % of the population died, whereas for gamma, 8.3 %; delta, 5.6 %; and omicron, 4.0 % of the population died (Table 3)

**Table 3:** Outcomes regarding four VOC.

Outcomes	Variants n (%)						
	Wild-type	Gamma	aOR (95 % CI)	Delta	aOR (95 % CI)	Omicron	aOR (95 % CI)^a^
ICU admission	1171/3302 (35.5 %)	943/2607 (36.2 %)	1.07 (0.96–1.19)	263/726 (36.2 %)	1.17 (0.98–1.39)	1107/3428 (32.3 %)	0.99 (0.89–1.10)
Intubation	472/3117 (15.1 %)	337/2497 (13.5 %)	0.91 (0.78–1.06)	96/713 (13.5 %)	1.01 (0.79–1.28)	340/3212 (10.6 %)	0.78 0.67–0.91)
Death	335/3271 (10.2 %)	217/2612 (8.3 %)	0.84 (0.70–1.01)	41/734 (5.6 %)	0.60 (0.43–0.85)	141/3501 (4.0 %)	0.43 (0.35–0.53)

a aOR, OR adjusted for ethnicity, age, cardiac and hepatic disease.

When comparing only the delta and omicron variants, using delta as the baseline, dyspnea (aOR = 1.30, 95 % CI 1.09–1.56) presented as a risk factor and diarrhea as a protective factor (aOR = 0.71, 95 % CI 0.61–0.97) for infection by the omicron variant. However, in the outcome analysis, intubation was more frequent in delta infections. (Table 4, Table 5).

**Table 4:** Symptoms regarding delta and omicron.

Symptoms	Variants n (%)			
	Delta^a^	Omicron	OR (95 % CI)	aOR (95 % CI)
Fever	563/714 (78.9 %)	2703/3339 (81.0 %)	0.80 (0.66–0.98)	1.13 (0.90–1.36)
Cough	583/719 (81.1 %)	2699/3305 (81.7 %)	1.03 (0.84–1.27)	1.00 (0.81–1.24)
Dyspnea	367/654 (56.1 %)	1791/2935 (61.0 %)	1.22 (1.03–1.45)	1.30 (1.09–1.56)
Respiratory discomfort	394/636 (61.9 %)	1936/2999 (64.6 %)	1.11 (0.93–1.33)	1.11 (0.92–1.32)
Desaturation	314/625 (50.2 %)	1559/2858 (54.5 %)	1.18 (0.99–1.41)	1.18 (0.99–1.41)
Diarrhea	114/554 (20.6 %)	438/2570 (17.0 %)	0.79 (0.62–0.99)	0.71 (0.61–0.97)
Vomit	104/545 (19.1 %)	546/2599 (21.0 %)	1.12 (0.89–1.42)	1.10 (0.87–1.40)

a Baseline/aOR, Adjusted for ethnicity, age, and cardiac and hepatic diseases.

**Table 5:** Outcomes regarding delta and omicron.

Outcomes	Variants n (%)			
	Delta^a^	Omicron	OR (95 % CI)	aOR (95 % CI)
ICU admission	263/726 (36.2 %)	1107/3428 (32.3 %)	0.83 (0.71–0.99)	0.85 (0.71–1.01)
Intubation	96/713 (13.5 %)	340/3212 (10.6 %)	0.76 (0.59–0.96)	0.77 (0.60–0.99)
Death	41/734 (5.6 %)	141/3501 (4.0 %)	0.70 (0.49–1.01)	0.71 (0.49–1.03)

a Baseline/Aor, OR adjusted for ethnicity, age, and cardiac and hepatic diseases.

## Discussion

Our study highlights that children under 2-years of age are also at risk of severe COVID-19 and that symptoms may depend on the COVID-19 variant they are infected with. Additionally, our study suggests changes in public health policies to corroborate the importance of vaccination for the pediatric population, especially during pandemic times.

Most of the study population was infected with the wild-type and omicron variants. The gamma variant is associated with severe COVID-19 symptoms, although this VOC is not highly virulent in countries other than Brazil. Corroborating other studies, there was no significant difference between the sexes in relation to case distribution and outcomes.^3^^,^^6^^,^^8^ Most of the individuals < 2-years of age stricken by COVID-19 were between 1 and 6-months.^18^ The notion that this population is the most vulnerable to infection concerns since there is presently no vaccination coverage. However, in this study, there was no knowledge regarding maternal vaccination and its impact on infectiveness and case severity in children aged <6-months. A study published in the *New England Journal of Medicine* in 2022 analyzing 537 infants (<6-months-old) concluded that maternal vaccination with two doses of the mRNA vaccine was associated with a lower risk of hospitalization and severe illness by SARS-CoV-2.^18^ These data highlight the importance of encouraging maternal vaccination and aiming for infant protection. In Brazil, pregnant women and infants have low vaccination rates, primarily because of disinformation and fear of adverse effects.

Several studies indicated that the omicron variant is more infectious, although less likely to cause severe disease, which corroborates our results that most of the population was affected by omicrons; this variant was a protective factor for the necessity of intubation and death.^7^^,^^9^^,^^19^^,^^20^ When exclusively comparing delta and omicron, infection by the latter was also associated with a lower risk of intubation. Thus, although omicrons appear to be infectious and account for a larger proportion of cases, deltas are associated with more severe outcomes, such as intubation. Additionally, some differences in the findings between these variants and the results with no significance in this study can be accounted for by the different number of cases found for each variant; the omicron variant had at least four times more cases than the delta variant. A study published by *Clinical Infectious Disease* analyzing children and adolescents <18-years of age with >1735 cases of delta variants and 32,635 cases of omicrons identified that 15.7 % of children infected with delta variants had moderate disease, whereas 2.2 % of omicron infections evolved with moderate disease. They also identified that the population aged <6-years was at a higher risk of hospitalization, need for intensive care unit admission, and mechanical ventilation.^10^

Among the four main VOCs studied, the wild-type variant was associated with the greatest need for intubation and death. These results may also be associated with the period during which the variant was most prevalent. The wild-type variant peaked at the beginning of the COVID-19 pandemic when little information was available regarding treatment and disease management.

Our results corroborate the literature regarding the most common symptoms in patients aged <2-years, independent of the VOC: fever and cough.^7^^,^^18^^,^^19^ Another meta-analysis of pediatric patients identified fever in 51 % of patients and cough in 41 %, regardless of the variants being studied.^20^

In our study, we analyzed respiratory discomfort, desaturation, and dyspnea in different VOC prevalence periods to better understand case severity. In some cases, dyspnea and desaturation were present; however, the patient could have been treated with an oxygen mask, for example, not requiring intubation. Nevertheless, this reflects the severity when comparing the variants.

Children are at lower risk of developing comorbidities. The literature highlights that for children aged <2-years, chronic lung disease, neurologic disease, cardiovascular disease, and prematurity are associated with severe COVID-19 cases.^21^ In this study, comorbidities seemed to affect different VOC, cardiac, and hepatic diseases. No analysis of prematurity has been conducted.

At the beginning of the pandemic, children and adolescents were thought to be less susceptible and associated with milder symptoms of COVID-19, which initially did not grant this population open access to vaccination, as it was understood that the population was not a priority with regard to the risk of infection. The notion that children are less susceptible to moderate infection is associated with the expression of angiotensin-converting enzyme-2. Angiotensin-converting enzyme-2 is the primary receptor for SARS-CoV-2 and appears to have a protective role in limiting angiotensin-2 mediators. These mediators have been associated with an increase in inflammation of pulmonary vessels, and angiotensin-converting enzyme-2 is known to decrease with age.^22^^,^^23^ However, independent of the pathophysiology, with the presence of new VOC and the initial limited access to vaccination, severe infection was identified in children, especially those below 6-years of age.^9^ This study highlights the clinical presentations in children between different VOCs and the importance of understanding that this population is at high risk for moderate to severe cases of COVID-19. During pandemic scenarios, it is important to include vulnerable groups in vaccine studies. Pregnant women and children were excluded from the initial studies, thereby delaying the start of vaccination for this segment of the population despite the exceptional nature of the moment.

## Declaration of competing interest

The authors declare no conflicts of interest.
